# Characterization of Chemosynthetic Microbial Mats Associated with Intertidal Hydrothermal Sulfur Vents in White Point, San Pedro, CA, USA

**DOI:** 10.3389/fmicb.2016.01163

**Published:** 2016-07-27

**Authors:** Priscilla J. Miranda, Nathan K. McLain, Roland Hatzenpichler, Victoria J. Orphan, Jesse G. Dillon

**Affiliations:** ^1^Department of Geological Sciences, California State University, Long Beach, Long BeachCA, USA; ^2^Department of Biological Sciences, California State University, Long Beach, Long BeachCA, USA; ^3^Division of Geological and Planetary Sciences, California Institute of Technology, PasadenaCA, USA

**Keywords:** hydrothermal vents, microbial mat, *Desulfuromusa*, sulfur-cycling, sulfate reduction rates, pyrosequencing, fluorescence *in situ* hybridization (FISH)

## Abstract

The shallow-sea hydrothermal vents at White Point (WP) in Palos Verdes on the southern California coast support microbial mats and provide easily accessed settings in which to study chemolithoautotrophic sulfur cycling. Previous studies have cultured sulfur-oxidizing bacteria from the WP mats; however, almost nothing is known about the *in situ* diversity and activity of the microorganisms in these habitats. We studied the diversity, micron-scale spatial associations and metabolic activity of the mat community via sequence analysis of 16S rRNA and *aprA* genes, fluorescence *in situ* hybridization (FISH) microscopy and sulfate reduction rate (SRR) measurements. Sequence analysis revealed a diverse group of bacteria, dominated by sulfur cycling gamma-, epsilon-, and deltaproteobacterial lineages such as *Marithrix, Sulfurovum*, and *Desulfuromusa*. FISH microscopy suggests a close physical association between sulfur-oxidizing and sulfur-reducing genotypes, while radiotracer studies showed low, but detectable, SRR. Comparative 16S rRNA gene sequence analyses indicate the WP sulfur vent microbial mat community is similar, but distinct from other hydrothermal vent communities representing a range of biotopes and lithologic settings. These findings suggest a complete biological sulfur cycle is operating in the WP mat ecosystem mediated by diverse bacterial lineages, with some similarity with deep-sea hydrothermal vent communities.

## Introduction

Hydrothermal vent ecosystems are considered biogeochemical hotspots due to their unique physico-chemical conditions. The variable geochemistry of vents produces distinct biotopes (Olenin and Ducrotoy, 2006), which select for unique microbial communities (Kelley et al., 2002; Kormas et al., 2006; Perner et al., 2007; Nakamura et al., 2009; Campbell et al., 2013). Many vents support chemosynthetic microbial mat populations (Reysenbach and Shock, 2002; Nakamura et al., 2009; Emerson and Moyer, 2010) dominated by sulfide-oxidizing bacteria (SOxB) (Brazelton et al., 2006; Hügler et al., 2010; Flores et al., 2011; Jaeschke et al., 2012; Fleming et al., 2013).

Common phylotypes identified from these studies are mat forming, sulfur-oxidizing Epsilonproteobacteria (e.g., *Sulfurovum, Sulfurimonas*) and Gammaproteobacteria, especially filamentous forms responsible for the visually conspicuous nature of the white-colored microbial mats (e.g., *Beggiatoa, Thiothrix*) (Muyzer et al., 1995; Crépeau et al., 2011; Yamamoto and Takai, 2011; Kato et al., 2012). In addition to SOxB, sulfate-reducing bacteria (SRB), especially members of the Deltaproteobacteria, are common in hydrothermal sites (Jannasch et al., 1988; Houghton et al., 2007; Frank et al., 2013). Endogenous sulfate reduction rates (SRR) at hydrothermal environments are typically higher (Weber and Jørgensen, 2002; Frank et al., 2013) than cold marine sediments (Thamdrup and Canfield, 1996; Ferdelman et al., 1997; Weber and Jørgensen, 2002; Frank et al., 2013). This raises the possibility of a complete biological sulfur cycle mediated by partnerships between oxidative and reductive sulfur bacteria. Tightly coupled (cryptic) sulfur cycling, where physically associated oxidative and reductive metabolic types co-exist, has been identified in phototrophic microbial mats and consortia (Fike et al., 2008; Wilbanks et al., 2014), and has recently been demonstrated in a companion investigation to this one (Dawson et al., 2016) in the White Point (WP) chemosynthetic vent mat communities found in the Palos Verdes (PV) hydrothermal vent field in San Pedro, CA, USA. However, it is not clear how significant a role biological sulfur/sulfate reduction plays in the WP mats.

Diverse, sulfur-cycling microbial mats have been observed in a range of marine, hydrothermal settings including mid-ocean ridges (Gerasimchuk et al., 2010; Lanzén et al., 2011; Urich et al., 2014), back arc spreading centers (Kato et al., 2009) and arc volcanoes (Emerson and Moyer, 2002; Murdock et al., 2010). The composition and structure of the chemoautotrophic population has been shown to vary across hydrothermal systems with varying geochemical energy sources (Desbruyères et al., 2001; Kelley and Shank, 2010; Amend et al., 2011; Flores et al., 2011; Nakamura and Takai, 2014) and lithologic composition (Baker and German, 2004). Most identified hydrothermal ecosystems in the ocean can be characterized within five major lithologic classifications (ultramafic, mafic/basaltic, andesitic, felsic or sediment) (Buatier et al., 1995; Embley et al., 2007; McCaig et al., 2007; Pašava et al., 2007; Tivey, 2007; Kakegawa et al., 2008; Zielinski et al., 2011) or as hybrid systems (e.g., basalt-sediment) (Nakamura et al., 2009; Amend et al., 2011).

Although deep-sea hydrothermal vents have been studied for decades (Corliss et al., 1979; Jannasch and Mottl, 1985; Tunnicliffe et al., 1986), their remote nature makes investigations challenging and expensive. Shallow-sea hydrothermal vent systems such as those identified from Italy, Greece and Mexico (Sievert et al., 1999; Amend et al., 2003; Forrest et al., 2005; Rusch et al., 2005; Price et al., 2013) as well as PV in the USA (Jacq et al., 1989; Kalanetra et al., 2004), represent more easily accessed analogs to study chemosynthetic microbial communities, although it is largely unknown how they compare to their deep-sea counterparts (Tarasov et al., 2005) where environmental conditions such as light, pressure, temperature and geochemistry are known to differ. This study focuses on the microbial mats that inhabit the intertidal region of WP, a hybrid basalt-sediment-hosted system in the PV hydrothermal vent field. Past studies of the WP mats have been limited, using microscopy, fatty acid characterization and cultivation approaches to investigate the large, filamentous sulfur-oxidizing Gammaproteobacteria found there (Jacq et al., 1989; Kalanetra et al., 2004). Another study investigated grazing of the mats by abalone (Stein, 1984); however, almost nothing is known about the overall diversity of the microbial mat community, the microorganisms involved or their biogeochemical interactions. Here, we combined molecular sequencing, fluorescence *in situ* hybridization (FISH) and SRR activity measurements to characterize this shallow-sea hydrothermal vent ecosystem.

## Materials and Methods

### Sample Collection

**FIGURE 1:**
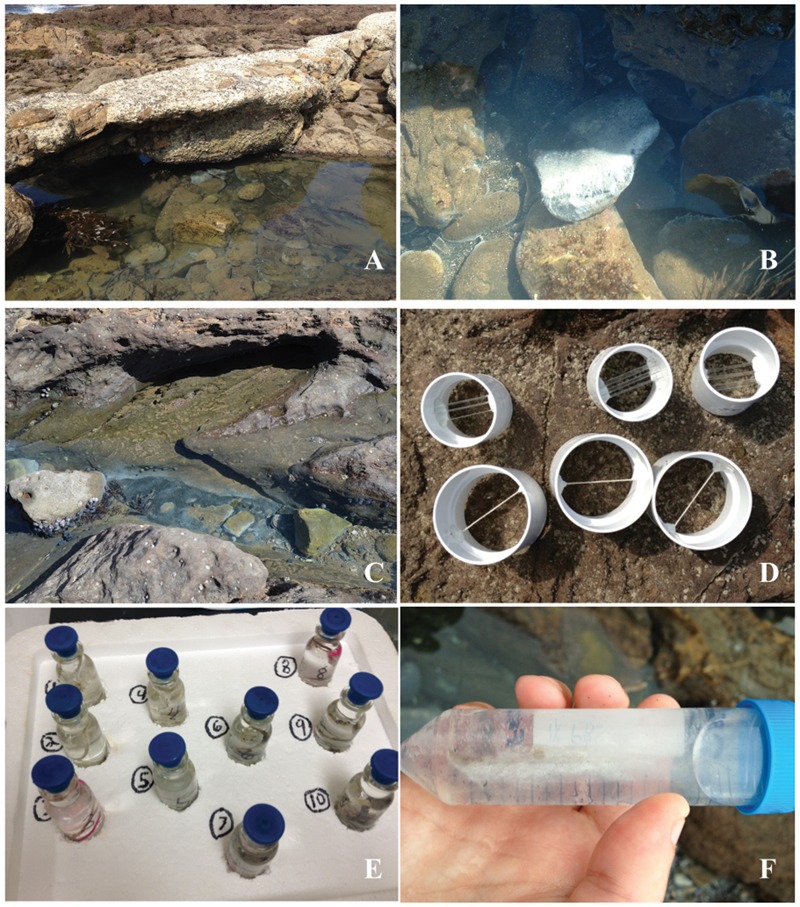
**Photographs of WP rocky intertidal hydrothermal field site and collection apparati. (A)** Field site, **(B)**, white bacterial mats and streamers covering rocks and **(C)** associated blackened (sulfidic) sediment patches. **(D)** PVC tubes containing natural fiber strings and glass microscope slides prior to deployment at field site. **(E)** Field collected samples of colonized strings in stoppered bottles and **(F)** colonized glass microscope slide in conical tube.

Microbial mat samples were collected repeatedly over 2 years (2012–2013) from the WP rocky intertidal hydrothermal vent field of the PV Peninsula (33.7159° N, 118.319° W) (**Figure 1A**) using a range of methods for different analyses. Replicate samples (e.g., duplicate sequencing) were always collected from individual rocks from within the same intertidal pool at WP (**Figure 1A**). Intertidal WP vents emit warm (∼28°C), sulfide-rich water (up to 650 μM/L) (Dawson et al., unpublished). White-colored microbial mats and streamers indicate diffuse venting in rocky substrates (**Figures 1A,B**); while over sediments, mats and blackened (sulfidic) sediment patches (**Figure 1C**) indicate venting. In the field, mat samples for DNA extraction were collected from colonized rock by scraping with a sterile razor and transferred into sterile 1.5 mL tubes. Duplicate rock scrapings were collected in June, 2012, for Sanger sequencing and two more rock scrapings were collected in February, 2013, for pyrosequencing (see below). All samples for molecular analyses were immediately frozen on dry ice for transport, then stored at -80°C in the laboratory until further analysis.

Natural fiber strings and glass microscope slides were mounted inside PVC pipes using water-resistant epoxy putty (J-B Weld, Sulfur Springs, TX, USA; **Figure 1D**) and deployed near the vents for 3 weeks at a time in August, October, and December 2013. String samples were collected for SRR and sealed with the hydrothermal eﬄuent in 15 mL serum bottles (**Figure 1E**; Bellco, Vineland, NJ, USA), then transported to the lab for incubation (see below). Mat samples that colonized deployed glass slides in Aug. were preserved for FISH by placing the slide into 50 ml conical tubes (BD Biosciences, Franklin Lakes, NJ, USA) containing 1X phosphate-buffered saline (PBS) (130 mM sodium chloride, 10 mM sodium phosphate buffer [pH 7.2]) and then kept on ice prior to fixation with 4% paraformaldehyde (PFA) (**Figure 1F**) in the laboratory within 1.5 h (Daims et al., 2005).

### Sulfate Reduction Rate Assays

A preliminary, time course experiment was conducted to determine a suitable incubation time for microbial sulfate reduction. This experiment indicated that SRR increased linearly over a 96 h period (Slope: 116.04, Intercept: 950.47, *R*^2^= 0.86229); therefore, 72 h incubations were used for subsequent experiments. On two different dates (September, December 2013) replicate experiments were performed. For each, the collected colonized strings (*n* = 5–6 sample replicates) were placed in 15 ml serum bottles containing 5 ml of hydrothermal eﬄuent, were injected with 0.37 MBq (10 μCi) of carrier-free Na_2_[^35^SO_4_] (American Radiolabeled Chemicals, St. Louis, MO, USA) and incubated at room temperature for 72 h. In addition, two control bottles were prepared as above with the addition of sodium molybdate (Mo; 20 mM) to inhibit microbially mediated sulfate reduction (Oremland and Capone, 1988). Two additional negative controls were killed with 20% zinc acetate and 37% formaldehyde immediately following the Na_2_[^35^SO_4_] addition. Following the 72 h incubation, reactions in the live and Mo control samples were terminated in the same manner. The samples were centrifuged at 3,220 *g* for 10 min. Pelleted mat samples were processed following a slightly modified version of the passive extraction procedure of Ulrich et al. (1997), adapted from the single-step chromium reduction method (Fossing and Jørgensen, 1989). The supernatant (unreduced sulfate fraction) was decanted into 15 ml conical tubes and stored at room temperature until analysis with a scintillation counter (LSC 6500, Beckman Instruments, Irvine, CA, USA). All oxygen-sensitive procedures were conducted in an anaerobic chamber (Coy Laboratory Products Inc., Grass Lake, MI, USA). SRR were calculated following the equation of Fossing and Jørgensen (1989).

### DNA Extraction and PCR Amplification, Cloning and Sequencing of 16S rRNA and *aprA* Genes

Total genomic DNA was isolated from 0.5 g of each of the rock scrapings described above in Sample Collection (2 for Sanger, 2 for Pyrosequencing) using the FASTDNA SPIN Kit for Soils (MP Biomedicals, Solon, OH, USA) according to the manufacturer’s instructions. Two of these independent extracts were used for bacterial 16S rRNA gene PCR amplifications performed using the GM3F and GM4R (Muyzer et al., 1995) primer pair in 50 μL reactions. Reaction mixtures contained 10 μL 5X PCR Buffer, 5 μL 25 mM MgCl_2,_ 0.5 U of GoTaq DNA Polymerase (Promega, Madison, WI, USA), 2.5 μL of 0.4% (w/v) Bovine Serum Albumin, 2.5 μL of each 10 μM primer (Eurofins MWG Operon, Huntsville, AL, USA), 1.5 μL of 10 mM dNTP mixture (Promega), and ∼20 ng of extracted nucleic acids as template. PCR was performed with a Mastercycler Pro Gradient PCR machine (Eppendorf, Hauppauge, NY, USA) under the following conditions: 5 min initial denaturation at 94°C, followed by 34 cycles of denaturation (94°C for 30 s), annealing (53°C for 30 s), and elongation (72°C for 90 s). Amplification was completed by a final elongation step at 72°C for 10 min. Additional PCR reactions using the Desulfobacteraceae-specific 16S rRNA gene DSS-658 probe as a reverse primer (to assess taxonomic specificity of this probe) and primers targeting the *apr*A gene were performed as above, except with use of the following forward and reverse primer sets: GM3F/DSS-658 (Manz et al., 1998) and the AprA-1-FW/AprA-10-RV (Meyer and Kuever, 2007) respectively. PCR products were visually analyzed by electrophoresis on 1% agarose gels run in 1X TAE buffer to verify correct amplicon size.

Positive 16S rRNA (both bacterial GM3/GM4 and Desulfo bacteriaceae-specific GM3/DSS-658) and *apr*A gene amplicons were cloned and sequenced as previously described (Dillon et al., 2013). Sanger sequences were submitted to Genbank and have been assigned accession numbers KX422076 – KX422101 for *aprA* and KX422102 – KX422190 for 16S rRNA genes.

### Sanger Sequence Analyses

The nucleotide sequence data from 16S rRNA and *aprA* gene Sanger sequences were trimmed and manually edited using 4Peaks Software^1^. Chimera detection for 16S rRNA gene sequences was performed using Mallard v.1.0 software (Ashelford et al., 2006) and putative chimeras confirmed by analyses with Pintail (Ashelford et al., 2005) were removed. Non-chimeric, full-length 16S rRNA gene sequences were aligned using the SINA aligner (Pruesse et al., 2012), imported into ARB software v5.2 (Ludwig et al., 2004) and manually refined with reference to close phylogenetic relatives. Full-length *apr*A sequences were initially aligned to all available gene sequences in the NCBI website using Clustal X (Larkin et al., 2007), and then imported into a custom-created ARB database. Custom lane masks of aligned sequences (both 16S rRNA and *apr*A genes) were created excluding hypervariable regions (16S rRNA gene sequences) and ambiguous nucleotide positions common to all sequences. A total of 1,185 (16S rRNA) and 1,273 (*apr*A) gene nucleotide positions, respectively were used to create maximum likelihood trees via the Blackbox RaxML tool on CIPRES Science Gateway v.7.2 (Miller et al., 2010). 1,000 bootstrap pseudo-replications, or fewer if stopped using the automated MRE bootstrapping criterion (e.g., 450, bacterial 16S rRNA gene tree), were performed.

Non-redundant DSS-658 16S rRNA gene sequences were first added to the full-length 16S rRNA gene tree using the parsimony tool in ARB to identify nearest relatives. Then a maximum likelihood tree including unique DSS-658 and relatives was constructed as above using 584 nucleotide positions.

### 16S rRNA Gene Pyrotag Analyses

Additional 16S rRNA gene pyrotag sequencing using the 530F/1100R primer pair (Lane, 1991; Dowd et al., 2008) was performed on two additional independent mat sample extracts (see above) by Research and Testing Laboratory (RTL), (Lubbock, TX, USA). These bacterial 16S rRNA gene 454 pyrosequences were denoised using RTL protocols and chimera-checked by the *de novo* method using UCHIIME (Edgar et al., 2011) with all low quality and possibly chimeric sequences removed. The remaining sequences were sorted and clustered into OTU clusters with 99% identity (1% divergence) using USEARCH (Edgar, 2010). These results were checked against the NCBI database (Altschul et al., 1997) using BLASTN+. Based upon the BLASTN+-derived sequence identities, the sequences were classified at the appropriate taxonomic level. More information on the RTL data analysis methodology can be found at http://www.researchandtesting.com. Alpha diversity was analyzed through rarefaction curves and both the Shannon–Weaver and the Simpson diversity indices were calculated. Beta diversity comparisons were performed in QIIME (Caporaso et al., 2010) using a Monte Carlo procedure. These sequences have been submitted to the SRA of NCBI and have been assigned project number SRP076744.

### Comparative Hydrothermal Vent Dataset Analyses

16S rRNA gene pyrosequencing data from a total of 13 publically available datasets (NCBI) from a range of hydrothermal environments/biotopes were compared to our two 454 libraries (**Table 1**). Sequences used were generated using commonly used V4-V6 region primer sets and had at least 100 bp overlap with our 530F-1100R dataset. The combined sequence datasets were uploaded and analyzed using the QIIME software pipeline (Caporaso et al., 2010). All hydrothermal microbial community datasets were rarefied to 2,500 sequences and randomized to eliminate sampling size bias among datasets in the analysis. UniFrac (Lozupone and Knight, 2005) distance matrices were calculated using both weighted and unweighted parameters in QIIME and exported for analysis in PRIMER v6.2 (Primer-E Ltd., Plymouth, UK). Primer was used to construct non-metric multidimensional scaling (MDS) plots and to perform analyses of similarity (ANOSIM) community comparisons among the vent pyrotag datasets. Comparisons were made after coding sequence datasets based on their associated vent parameters including depth (shallow, deep), system lithology (basalt, andesite, basalt-andesite hybrid) and hydrothermal biotope (vent fluids, mats, sediments + mats, sulfide chimneys and non-sulfide chimneys). Hydrothermal microbial communities were categorized as either above (shallow) or below (deep) 200 m depth, a cut-off previously used in describing hydrothermal systems (Prol-Ledesma et al., 2005; Tarasov et al., 2005).

**Table 1:** Hydrothermal systems used for comparative analysis in this study.

Region	Location	Study site	Depth	Geologic setting	System lithology	Biotope	References
North Pacific	Juan De Fuca-	Needles	Deep	MOR	Sediment	Sulfide Chimney/Deposits	Frank et al., 2013
	Middle Valley	Dead Dog	Deep	MOR	Sediment	Sulfide Chimney/Deposits	Frank et al., 2013
		Chowder Hill	Deep	MOR	Sediment	Sulfide Chimney/Deposits	Frank et al., 2013
	Mariana Arc	Nikko	Deep	Arc Volcano	Andesite	Hydrothermal fluids	Embley et al., 2007;
							Huber et al., 2010
		NW Eifuku	Deep	Arc Volcano	Basalt	Hydrothermal fluids	Embley et al., 2007;
							Huber et al., 2010
		Daikoku	Deep	Arc Volcano	Andesite	Hydrothermal fluids	Embley et al., 2007;
							Huber et al., 2010
		NW Rota 1	Deep	Arc Volcano	Basalt-Andesite	Hydrothermal fluids	Embley et al., 2007;
							Huber et al., 2010
		Forecast	Deep	Arc Volcano	Basalt	Hydrothermal fluids	Embley et al., 2007;
							Huber et al., 2010
	Okinawa Trough	Kueishantao	Shallow	BASC	Andesite	Hydrothermal fluids	Tang et al., 2013
Mediterranean	Hellenic Arc	Kolumbo	Shallow	Arc Volcano	Felsic	Sulfide Chimney/Deposits	Kilias et al., 2013
Arctic	Knipovich Ridge	Loki’s Castle	Deep	MOR	Basalt	Sulfide Chimney/Deposits	Jaeschke et al., 2012
	Jan Mayen	Troll Wall	Deep	MOR	Basalt	Sediments with mats	Lanzén et al., 2011
North Atlantic	Mid-Atlantic Ridge	Rainbow	Deep	MOR	Ultramafic	Sulfide Deposits	Flores et al., 2011

*MOR, Mid-Ocean Ridge; BASC, Back-Arc Spreading Center.*

### Fluorescence *In Situ* Hybridization

Field-colonized slides were washed twice in 1X phosphate buffered saline (PBS) before being fixed by immersion in freshly prepared 4% PFA solution in the field and placed on ice for transport to the laboratory. After 3 h on ice, the fixed slides were washed again in 1X PBS to remove residual PFA and then placed in 1X PBS: 96% ethanol (v:v) and stored at -20°C. FISH was performed following established protocols (Daims et al., 2005) using fluorescently labeled, group-specific oligonucleotide probes (**Table 2**). Hybridizations were performed using 1.5 ng μL^-1^ of HPLC-purified probe (Eurofins MWG Operon) with a buffer containing 0.01% sodium dodecyl sulfate and 35% formamide (Fisher Scientific) for 3 h at 46°C followed by a washing step at 48°C for 10 min. Washed and dried slides were counter stained with DAPI (1 μg mL^-1^) and mounted with citifluor AF-1 antifadent (CitiFluor, Leicester, England). Microscopic observation and documentation was performed using an epifluorescence microscope under 630X magnification (BX51, Olympus America Inc., Melville, NY, USA), fluorescent images were analyzed using ImageJ (Schneider et al., 2012).

**Table 2:** Probes used in this study.

Probe	Target group		
EUB338-I	Most (90%) Bacteria	GCT-GCC-TCC-CGT-AGG-AGT	Amann et al., 1990
DELTA495A	Most deltaproteobacteria	AGT-TAG-CCG-GTG-CTT-CCT	Loy et al., 2002
GAM42A	Gammaproteobacteria	GCC-TTC-CCA-CAT-CGT-TT	Manz et al., 1998
BET42	Betaproteobacteria	GCC-TTC-CCA-CTT-CGT-TT	Manz et al., 1998
SRB385	Most desulfovibrionales	CGG-CGT-CGC-TGC-GTC-AGG	Amann et al., 1990
DSS-658^a^	Desulfobacteraceae	TCC-ACT-TCC-CTC-TCC-CAT	Manz et al., 1998
DSM651	Desulfuromusa spp.	CCT-CTC-CCA-TAC-TCA-AG	This study
DSM651 competitor	Desulfuromusa spp.	CCT-CTC-CCA-TAC-TCT-AG	This study

*^a^Used as probe and primer.*

To visualize *Desulfuromusa*-related 16S rRNA phylotypes via FISH, a sequence-specific oligonucleotide probe (DSM651; **Table 2**) was designed manually using the ARB software package (Ludwig et al., 2004). Since probe DSM651 was a modification of the DSS658 probe previously described (Manz et al., 1998), the same formamide concentration (35%) was used. Probe DSM651 targets all *Desulfuromusa* sequences obtained from the clone libraries, but has at least one mismatch to all other sequences in the ARB database (SSURef_NR99_115_SILVA_20_7_13_opt.arb). FISH assays for *Desulfuromusa* were conducted using a DSM651 competitor probe (one base altered) to minimize non-specific hybridization non-targeted genotypes (**Table 2**).

## Results

### White Point Microbial Mat Phylogenetic Diversity

**FIGURE 2:**
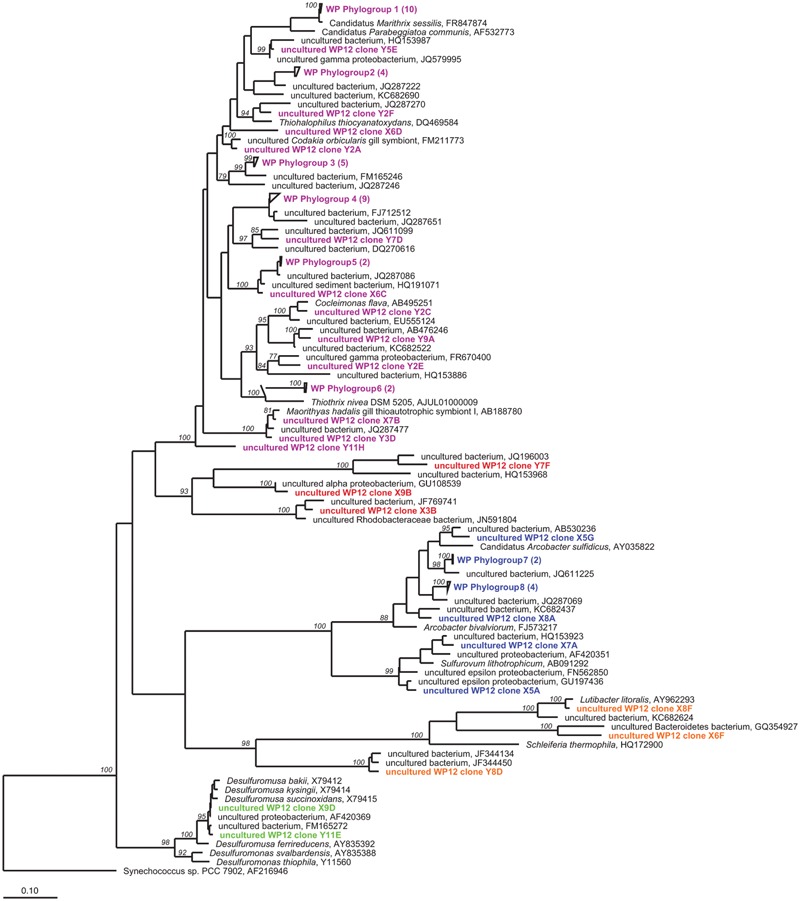
**Unrooted maximum-likelihood dendrogram of 16S rRNA gene sequences amplified from WP microbial mat community, aligned with closest relatives (black) using ARB software.** Colors indicate WP taxonomic groupings. Purple, gammaproteobacteria; red, alphaproteobacteria; blue, epsilonproteobacteria; orange, bacteroidetes, and green, deltaproteobacteria. Wedges indicate redundant sequences with number of sequences indicated in parentheses. Values at nodes indicate >75% bootstrap support. Scale bar shows a 10% estimated difference in nucleotide sequence positions.

Cloning and Sanger sequencing of the bacterial 16S rRNA genes yielded 79 non-chimeric nearly full-length sequences that were grouped into 62 unique OTUs (at 3% dissimilarity level). Taxonomic evaluation places these OTUs in five main clusters (**Figure 2**). The most diverse and abundant phylotype (71% of clones) were members of the sulfur-oxidizing order Thiotrichales within the Gammaproteobacteria. The next most abundant group (16%) of phylotypes branched within the Epsilonproteobacteria; 80 and 20% of which affiliated with members of the genera *Arcobacter* and *Sulfurovum*, respectively. The two deltaproteobacterial sequences were greater than 98% similar to cultured *Desulfuromusa kysingii* (Liesack and Finster, 1994). The remaining sequences were distributed between the Alphaproteobacteria and the Bacteroidetes (**Figure 2**).

**FIGURE 3:**
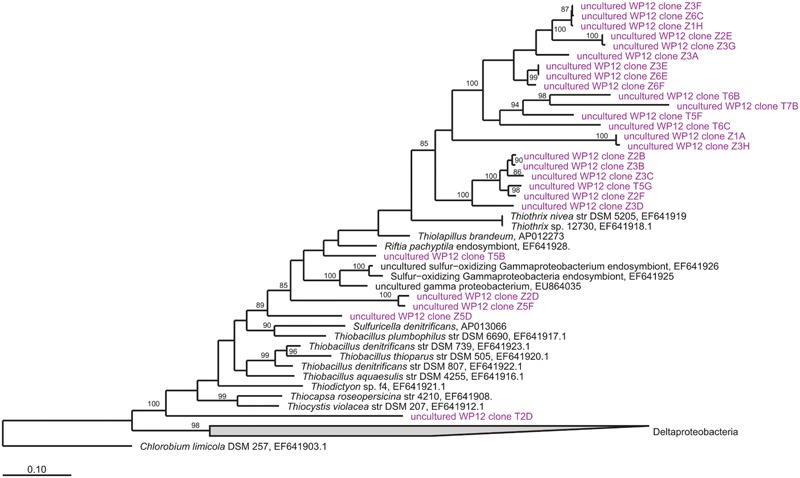
**Unrooted maximum-likelihood dendrogram of *apra*A gene sequences amplified from WP microbial mat community (purple), aligned with closest relatives (black) from a custom database.** Values at nodes indicate >85% bootstrap support. Scale bar shows a 10% estimated difference in nucleotide sequence positions.

In addition to the 16S rRNA gene sequencing, we also sequenced the functional gene, adenosine -5′-phosphosulfate reducates alpha subunit (*aprA*) gene to provide a targeted approach to investigate sulfur-cycling microorganisms in the WP mats. The *apr*A clone library resulted in a total of 39 clones with positive inserts, representing 26 novel, non-redundant genotypes all of which affiliated with oxidative gammaproteobacterial lineages (**Figure 3**). Despite the fact that this primer set is known to amplify reductive *aprA* gene copies, no deltaproteobacterial sequences were obtained.

**FIGURE 4:**
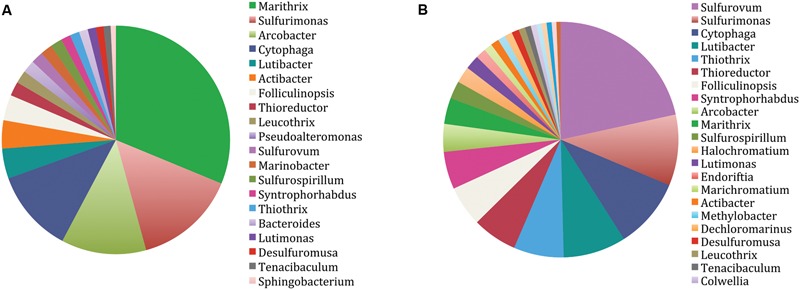
**Taxonomic distribution of the two WP microbial mat community pyrosequence datasets, **(A)** WP-1, **(B)** WP-2, displaying the relative (%) incidence of identified genera.** Total number of 1% dissimilarity OTUs assigned was **(A)** 28,802 and **(B)** 16,620.

**FIGURE 5:**
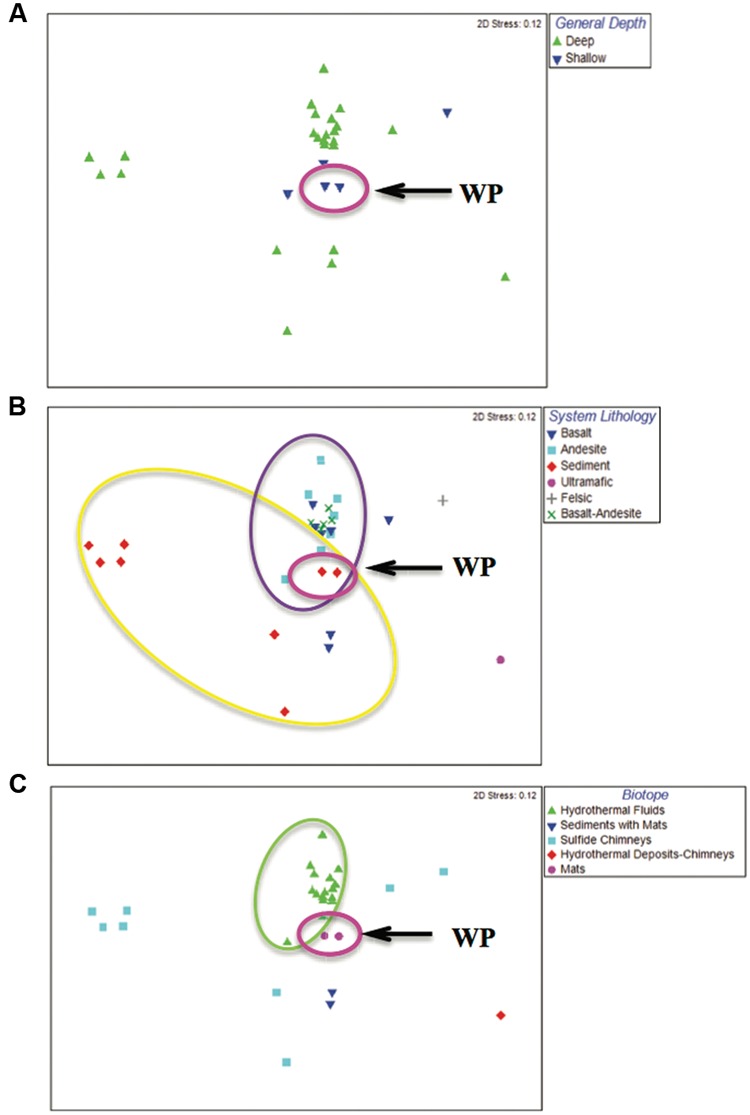
**Multidimensional scaling (MDS) plots showing community variation, coded (colored symbols) according to depth, system lithology and biotope.** Arrow and pink ellipse shows WP samples for all three panels, but does not denote statistical differences. **(A)** Depth. ‘Deep’ samples (>200 m) and ‘shallow’ samples (<200 m). **(B)** System lithology. Ellipses denote statistically significant communities as determined by ANOSIM (*p* < 0.005). Yellow denotes sediment-hosted sites; purple denotes Basalt, Andesite and Basalt-Andesite-hosted sites. **(C)** Biotope. Green ellipse denotes hydrothermal fluid sites that were significantly different from all others as determined by pair-wise ANOSIM comparisons (*p* < 0.01 for all).

Overall, the 454 pyrotag sequences displayed similar phylogenetic community composition patterns as the Sanger clones. The two pyrosequenced samples (WP-1, WP-2), generated 34,834 and 20,877 denoised, non-chimeric, high quality sequences, respectively and showed similar rarefaction curves indicating near-complete sampling (Supplementary Figure S1). Pyrotag sequences with abundances greater than 0.5% were clustered into 28,802 (WP-1) and 16,620 (WP-2) OTU’s at the 1% dissimilarity level (**Figure 4**). The Gammaproteobacteria and Epsilonproteobacteria classes accounted for approximately 69% (WP-1) and 66% (WP-2) of the sampled population. No significant differences in the overall community structure between the rarefied WP-1 and WP-2 datasets were detected (ANOSIM, *p* = 0.58). Monte Carlo analyses also found no significant differences between the samples when taken as a whole. However, differences in the dominant OTU (based on % composition) of the two libraries were observed, with uncultured Gammaproteobacteria of the *Marithrix* clade being most abundant in WP-1 and Epsilonproteobacteria most similar to *Sulfurovum* most abundant in the WP-2 library (compare **Figures 5A,B**). Overall, gammaproteobacterial pyrotags affiliated with the order Thiotrichales represented 31 and 11% of the communities, respectively. The most abundant members of the Thiotrichales in the two libraries had closest BLAST identities to *Marithrix sessilis* (Kalanetra et al., 2004) (WP-1) and to *Thiothrix nivea* (Stahl et al., 1987) (WP-2). *Marithrix sessilis* was the next most abundant gammaproteobacterium within WP-2 at 3%. Epsilon proteobacteria accounted for 32% (WP-1) and 43% (WP-2) of the library sequences. Interestingly, despite dominating the WP-2 library (19%), *Sulfurovum lithotrophicum*-like sequences only comprised 1.5% of the WP-1 library, which had greater prevalence of *Sulfurimonas*- (13%) and *Arcobacter*-like (10%) sequences (**Figure 4**).

Bacteroidetes, particularly members of the genus *Lutibacter*, represented the next most abundant group of pyrotag sequences recovered from the WP microbial mat samples (**Figure 4**). Members from three common classes of Bacteroidetes (Cytophaga, Flavobacteria, and Bacteroidia) accounted for roughly 24% (WP-1) and 22% (WP-2) of the sequences. Deltaproteobacteria accounted for 3% (WP-1) and 7% (WP-2) of the total number of pyrotags in the two WP microbial mat samples. The most abundant deltaproteobacterial genotype (1, 4% of the total 16S rRNA genes from the two pyrotag libraries, respectively) recovered was 99% similar to a deltaproteobacterial epibiont of the polychaete *Alvinella pompejana*, from deep-sea hydrothermal vents of the East Pacific Rise. As with the clone library, sequences affiliated with *Desulfuromusa* were detected, accounting for 30% (WP-1) and 12% (WP-2) of the Deltaproteobacterial pyrotags (∼1% of total sequences in ea. library). Among these, sequences with BLAST similarity to all four isolated species of the genus (*D. kysingii, D. succinoxidans, D. ferrireducens*, and *D. bakii*) (Liesack and Finster, 1994; Vandieken et al., 2006) were recovered. Additionally, sequences similar to a fifth, uncultured *Desulfuromusa* sp. were recovered from our samples. Minor additional phylotypes [cumulatively ∼3% (WP-1) and ∼4% (WP2) respectively] were identified as belonging to the Alpha- and Betaproteobacteria, Firmicutes, Tenericutes, Verrucomicrobia, and the Cyanobacteria (**Figure 4**).

Despite these differences noted above, the pyrotag sequence datasets had similar community richness. Rarefaction analysis revealed similar curves with high OTU richness from the two WP microbial mat samples (Supplementary Figure S1). Results of the Shannon–Weaver (H) and Simpson (D) diversity index calculations confirm the high levels of diversity within these two libraries (*H* = 8.67 and *D* = 0.95 for WP-1 and *H* = 9.51 and *D* = 0.98 for WP-2).

### Comparative Analyses of Hydrothermal Microbial Communities

The 454 pyrotag sequences identified from the WP microbial mat community were compared collectively with 13 hydrothermal vent microbial community studies for which data were available in Genbank (**Table 1**). MDS analysis showed that the WP hydrothermal microbial community plotted centrally among samples, but appeared closest to samples from the Okinawa Trough (Tang et al., 2013) (**Figure 5**). Due to low sample replication in our study and many of these published datasets, we could not perform pair-wise comparisons between the WP samples and others. However, when datasets were grouped based on depth (shallow, deep), system lithology (basalt, andesite, basalt-andesite hybrid) and hydrothermal biotope (vent fluids, mats, sediments + mats, sulfide chimneys and non-sulfide chimneys), patterns of community associations across datasets were revealed.

Analyses of similarity comparisons between depth categories did not reveal significant differences between the shallow-sea hydrothermal microbial communities and the deep-sea hydrothermal microbial communities (**Figure 5A**). However, coding of samples based on hydrothermal system lithology revealed significant differences (**Figure 5B**). ANOSIM results indicated that the sediment-associated vent sites (i.e., Juan de Fuca, WP) were significantly different from the andesite-, basalt- and the basalt-andesite-hosted sites (*p* < 0.005 for all comparisons). Felsic and ultramafic site samples (Flores et al., 2011; Kilias et al., 2013) occurred as outliers and plotted furthest from the central clusters, although they were not significantly different from the other igneous lithologies. Finally, coding samples according to their hydrothermal biotope also revealed significant differences, as samples from hydrothermal fluids were significantly different from samples collected from the other three biotopes (*p* < 0.01 for all), although the non-fluid biotope communities were not significantly different from each other (*p* > 0.05 for all) (**Figure 5C**).

### Fluorescence *In Situ* Hybridization

**FIGURE 6:**
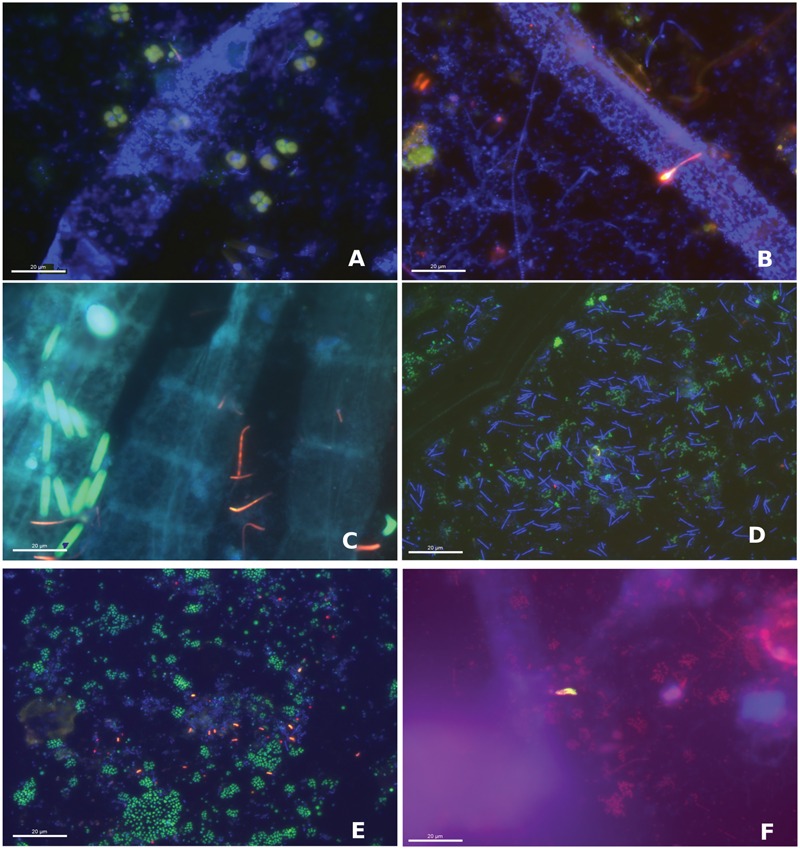
**Fluorescence *in situ* hybridization (FISH) images of the WP microbial mat community showing different fields of view from two colonized glass slides collected in June 2014 at 630X magnification. (A)** Large filament (blue) showing DAPI signal and tetrads of Gammaproteobacteria (green). **(B)** DAPI-stained filaments (blue) and Gammaproteobacteria (green) with chains, rods and cocci Deltaproteobacteria (red; Delta495). **(C)** Large filaments (blue) with rod-shaped Gammaproteobacteria (green) and chains of Deltaproteobacteria (red; DSS658). **(D)** Groups of gammaproteobacterial cocci (green) with DAPI-stained filaments (blue). **(E)** Gammaproteobacterial cocci (green), deltaproteobacterial curved rods (red, DSS658) and DAPI- stained cocci and filaments (blue). **(F)** Aggregates of Deltaproteobacteria (yellow; DSS658) with Gammaproteobacteria (red) and DAPI-stained filaments (blue).

We performed FISH on six colonized slides to visualize the spatial orientation and relative abundance of the microbial populations inhabiting the WP microbial mat community. Very large (∼30–40 μm diameter) *Marithrix*-like filaments were consistently visualized with DAPI during our study. However, we failed to demonstrate a positive probe signal with the GAM42A (used with its competitor Bet42a) gammaproteobacterial-specific probe (Manz et al., 1998) or the more general bacterial probe EUB338 (Amann et al., 1990) on these large filaments during any of our FISH experiments (**Figures 6A–C,F**). Overall, we observed lower relative abundances of deltaproteobacteria-positive cells compared to Gammaproteobacteria-positive cells. Cells successfully probed with the DELTA495A and DSS658 probes targeting Deltaproteobacteria and the Desulfobacteraceae, respectively, (Manz et al., 1998; Loy et al., 2002) included single-cells (mean length 1.7 μm, *n* = 20 cells) (1–2 μm length) and aggregates of cocci to short chains of cells (range 10–20 μm length, *n* = 20 chains) (**Figures 6B–D**). The SRB385 probe, which specifically targets members of the Desulfovibrionales (Amann et al., 1990) rarely produced a signal; when positive probing occurred it was to small (mean length 5 μm, *n* = 5) aggregates of coccoid cells (**Figure 6F**). In some cases, these SRP385-positive aggregates were found associated with the *Marithrix*-size filaments. Unlike the *Marithrix* filaments a variety of gammaproteobacterial single-cells, tetrads, rods and short (20 μm length) filaments were detected using the GAM42A probe (**Figures 6A,C–E**). Of note were GAM42A-positive clusters of cells that were consistently observed throughout our study (**Figure 6E**).

The specificity of the DSS-658 probe was checked by PCR and sequencing using the probe as a reverse primer. Among the 26 non-redundant phylotypes, the majority (69 %) fell within the Deltaproteobacteria, the majority of which were most closely related (>96% similarity) to cultured *Desulfuromusa* species (Liesack and Finster, 1994) (Supplementary Figure S2). However, the remaining 31% of clones were affiliated with members of the Epsilonproteobacteria and the Gammaproteobacteria, including SOxB phylotypes (Moyer et al., 1995; Gros et al., 1996; Takai et al., 2006a). This suggests that the DSS658 probe was likely not specific to Deltaproteobacteria for the WP mat.

**FIGURE 7:**
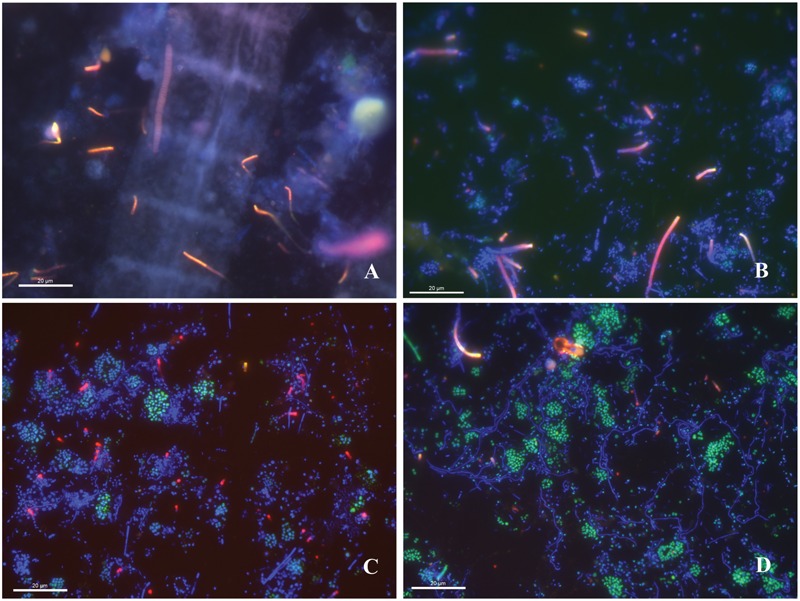
**Fluorescence *in situ* hybridization images of the WP microbial mat community at 630X magnification hybridized using the DSM-651 *Desulfuromusa*-specific probe. (A)** Large DAPI-stained filament (blue) and chains of *Desulfuromusa* rods (red). **(B)** DAPI-stained cells (blue) and *Desulfuromusa* (red) emerging from a central axis and chains. **(C)** DAPI-stained filaments and cocci (blue) with gammaproteobacterial cocci (green) and *Desulfuromusa* (red). **(D)** Gammaproteobacterial cocci clusters (green) with DAPI-stained filaments (blue) and *Desulfuromusa* cells (red).

Since *Desulfuromusa*-like sequences were abundant in the DSS-658 primer clone library (although still rare in the overall 16S rRNA community) and curved rods diagnostic of this group were observed in the DSS-658 FISH (**Figures 6C,E**), we decided to target this group specifically via FISH using a newly designed, highly specific *Desulfuromusa*-targeted probe (DSM651) to verify their importance and to identify their association(s) within the mat community. Probe DSM651 produced a signal on all slides with the prevailing cell morphology being short rods forming chains up to 30 μm in length (**Figure 7**). In some cases, multiple filaments appeared to originate from a central axis (**Figure 7B**). *Desulfuromusa* were also often found in association with Gammaproteobacteria, including the large *Marithrix*-like filaments (**Figure 7A**), as well as thinner filamentous forms and clusters of unicells.

### Sulfate Reduction Rates

**FIGURE 8:**
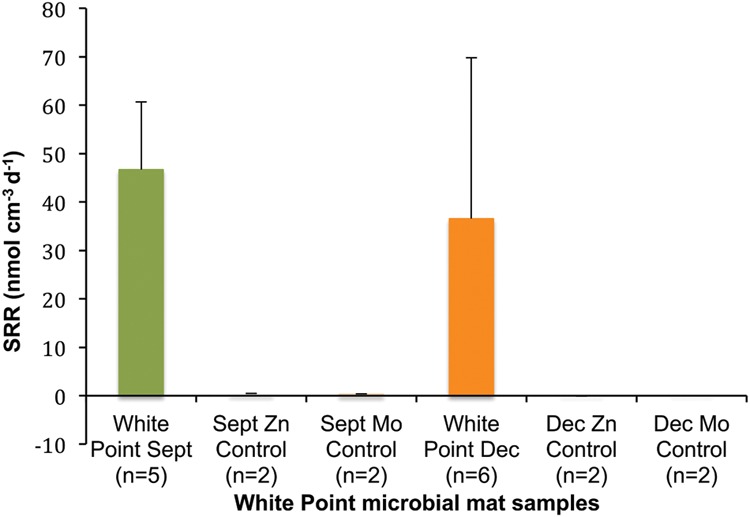
**Mean SRR for WP microbial mat samples.** Negative controls (Mo and kill) were always less than 1% of live rates (<1 nmol SO4 cm^-3^ day^-1^). Error bars = 1 *SD*.

Very similar rates were observed during two subsequent SRR experiments with WP mat samples in September and December 2013. During both field samplings, the mean SRR for the intertidal mats were significantly above zero (1-sample *T*-test, *p* < 0.05 for both) at 46.7 and 36.5 nmol SO_4_ cm^-3^ d^-1^, respectively (**Figure 8**). SRR rates measured from our sodium molybdate inhibited and Zn-formaldehyde killed controls were less than 1% of the rates measured in the live controls (<1 nmol SO_4_ cm^-3^ d^-1^).

## Discussion

Deep-sea hydrothermal systems have long been known to support chemosynthetic sulfur-cycling microbial populations (Corliss et al., 1979; Karl et al., 1980; Jannasch and Mottl, 1985), although relatively fewer studies have been performed on shallow hydrothermal vent systems (Sievert et al., 2000; Giovannelli et al., 2013; Tang et al., 2013; Wang et al., 2015). Based on results from previous studies of the WP sulfur vent microbial mat community (microscopy, fatty acid characterization and cultivation-dependent approaches) (Jacq et al., 1989; Kalanetra et al., 2004), we hypothesized that a sulfur-cycling consortium comprised of SOxB converting geologically derived vent sulfide to elemental sulfur and sulfate, and SRB converting oxidized, biologically derived sulfur species back to sulfide, would be present in the mats.

Our hypothesis was supported using multiple approaches to study the WP microbial mats. Both microscopy and gene sequencing revealed the importance of filamentous gammaproteobacterial sulfur-oxidizing lineages such as *Marithrix* and *Thiothrix*. *Marithrix*-like filamentous bacteria have also been detected in 16S rRNA gene surveys of mats associated with cold seeps in the Eastern Mediterranean and the Barents Sea in the Eastern Atlantic suggesting they may be common in lower temperature vent mats such as those found at WP (Heijs et al., 2005; Grunke et al., 2012). These large filamentous microorganisms did not hybridize with any of the general bacterial probes (e.g., EUB338), possibly due to the thick sheath enclosing the cells. However, the DAPI staining pattern, large size and distinct morphology is consistent with *Marithrix*. These conspicuous *Marithrix*-like filaments comprise a significant fraction of the biomass in these samples, and also appear to be a dominant member of the mat community based on *aprA* and 16S rRNA gene libraries. Remarkably, there were also dozens of novel phylotypes affiliated with the Thiotrichales detected using 16S rRNA as well as *aprA* gene sequencing. Other studies of shallow vents have found members of the Thiotrichales to be key members of the chemoautotrophic vent communities (Brinkhoff et al., 1999; Dawson et al., 2016). However, a number of studies in both shallow and deep-sea vents systems have found mesophilic Epsilonproteobacteria such as *Sulfurovum* and *Sulfurimonas* to be the most common groups of SOxB recovered by molecular means (Flores et al., 2012; Sylvan et al., 2012; Wang et al., 2015) and cultivation (Inagaki et al., 2003, 2004). Our 16S rRNA gene sequencing revealed several epsilonproteobacterial lineages closely associated with *Sulfurovum, Sulfurimonas*, and *Arcobacter*, three known groups of SOxB to be among the most abundant OTUs. Microbial sulfur oxidation pathways differ between epsilon- and gammaproteobacterial sulfur oxidizers; these differences have been hypothesized to represent distinct ecophysiological strategies, for example Epsilonproteobacteria are also known to be able to use sulfur and thiosulfate as alternative electron donors (Yamamoto and Takai, 2011). The prevalence of the two groups of sulfur bacteria may be related to the geochemical setting. For example, the predominance of Gamma- vs. Epsilonproteobacteria in molecular libraries has been found to vary with distance from the vent source, which may be due to varying tolerance to anoxia (Tang et al., 2013; Wang et al., 2015; Zbinden et al., 2015). Additionally, the influence of differences in vent geochemistry (largely driven by compositional differences of underlying rocks) on sulfur cyclers as well as other common vent groups has been noted in numerous studies (Huber et al., 2003; Schrenk et al., 2004; Kelley et al., 2005; Nakagawa et al., 2005; Campbell et al., 2006, 2013; Amend et al., 2011; Flores et al., 2011, 2012) although biogeographic isolation has also been identified as a potential contributing factor to variation in vent community structure (Huber et al., 2010).

The next most abundant OTUs recovered were affiliated with the phylum Bacteroidetes, which were dominated by lineages from the genus *Lutibacter*. Cultured relatives include *Lutibacter profundi*, a microaerophilic heterotroph, first isolated from a microbial mat on a black smoker chimney at Loki’s castle hydrothermal vent in the Arctic mid-ocean ridge (Le Moine Bauer et al., 2016). Members of the Bacteroidetes are typically rare in molecular surveys of deep-sea vent systems including Loki’s castle (Flores et al., 2012; Jaeschke et al., 2012), however, they have been previously described from shallow vent systems (∼10–20% of OTUs) (Sievert et al., 2000; Giovannelli et al., 2013; Wang et al., 2015). Members of the genus *Lutibacter* were not noted as being important in other shallow systems, suggesting a unique aspect to the WP mat community.

In contrast to the diverse SOxB proteobacterial lineages described above, we detected much lower relative sequence abundances and diversity of likely sulfate-/sulfur-reducing deltaproteobacterial lineages, representing 1–4% of the overall community. The most abundant groups of deltaproteobacterial 16S rRNA phylotypes were affiliated with the genus *Desulfuromusa* and an uncultured epibiont on *Alvinella pompejana* tubeworms, using cloning and Sanger sequencing and pyrosequencing, respectively. The importance of these groups contrasts with other deep-sea vent systems where other genera of Deltaproteobacteria such as *Desulfobulbus* and *Hippea* have been more commonly found (Flores et al., 2011, 2012). No Deltaproteobacteria were recovered using the *aprA* gene, which may be due to under sampling and the low abundance of SRB relative to the diverse SOxB phylotypes in the sample. Cultures of *Desulfuromusa* are only known to be sulfur reducers, not sulfate reducers (Liesack and Finster, 1994; Vandieken et al., 2006), so these mat microbes would not be predicted to possess *aprA* genes. Our FISH analyses using group-specific oligonucleotide probes also revealed low abundances of Deltaproteobacteria cells (∼2–5% of hybridized cells) compared to Gammaproteobacteria-positive cells (always greater than 20%). *Desulfuromusa* cells were occasionally observed to be physically associated with the large filamentous *Marithrix* – like filamentous bacteria suggesting that they may form epibiotic associations. This is reminiscent of previous reports of sulfate-reducing *Desulfonema* found in association with members of the ensheathed cyanobacterium *Coleofasciculus* (formerly *Microcoleus*) in hypersaline microbial mats (Fike et al., 2008). The *Desulfuromusa* may be metabolizing elemental sulfur produced by the SOxB.

The 16S rRNA gene-based analyses did reveal a relatively minor presence (<1% of both pyrotag libraries) of phylotypes related to sulfate-reducing Deltaproteobacteria (e.g., *Desulfomonile*-like sequences), suggesting the metabolic potential for a complete sulfur cycle within the WP mat. This potential was confirmed by our ^35^SO_4_^2-^ radiotracer measurements of SRR that measurable biological sulfate reduction occurs in the WP mats. These SRR are among the first reported from shallow hydrothermal sulfur-vent systems, and are comparable to those typically reported in cold, coastal marine sediments (Howarth and Jørgensen, 1984; Jørgensen and Bak, 1991; Jørgensen and Nelson, 2004) as well as rates measured in sediments from a high temperature, deep-sea hydrothermal vent (Jørgensen et al., 1992). Our SRR are likely to be underestimates of the total reductive sulfur metabolism in the WP mat system. The SRR assay relies on the reduction of the labeled sulfate substrate and would not measure activity for groups only capable of reducing elemental sulfur. Thus, sulfur reduction may be of greater significance in these mats compared to typical marine sediments due to the relative importance of groups like *Desulfuromusa*.

One goal of this study was to compare the microbial diversity from this warm, shallow WP hydrothermal vent field to its hard-to-access deep-sea counterparts. Comparisons with database sequences revealed many similar phylotypes in WP to those identified from deep-sea hydrothermal vents, especially a number of sulfur-cycling lineages (Moyer et al., 1995; Takai et al., 2006b; Murdock et al., 2010; Sylvan et al., 2013). When the WP pyrosequences were compared with other large hydrothermal vent 16S rRNA datasets, a number of trends with regards to vent system depth, lithology and biotope emerged. The two other shallow samples tended to cluster near the WP samples in our MDS plots; however, no significant differences were observed between shallow and deep communities via ANOSIM. This finding may have been impacted by the relative paucity of datasets from shallow systems compared to their deep-sea counterparts and the low number of sample replicates (see discussion of this study limitation below). Greater variation was observed for the deeper vent communities, potentially resulting from additional factors such as system lithology and biotope, factors that have been noted to affect hydrothermal vent communities in past studies (Desbruyères et al., 2001; Kato et al., 2010; Amend et al., 2011; Flores et al., 2011, 2012; Sylvan et al., 2013). Microbial communities from hydrothermal fluids were found to be significantly different from all other biotope categories and were least similar to the microbial mat/chimney-biofilm biotope communities including the WP samples. This is consistent with past findings that attachment-associated and biofilm populations were more diverse than planktonic fluid communities in hydrothermal environments at a deep-sea volcano (Huber et al., 2003; Edwards et al., 2005) and that mixing with surrounding seawater alters vent plume communities (Sheik et al., 2015). We also observed variation within a given biotope, which may be explained by system lithology. For example, differences were observed between the two clusters of sulfide chimneys in our MDS plots, which may reflect the fact that the sediment-hosted MOR samples support different communities than the two igneous sites (basaltic MOR and a felsic arc volcano) (Jaeschke et al., 2012; Kilias et al., 2013). Overall, our ANOSIM comparisons found communities from sediment-hosted vents were significantly different from all communities found on andesite- and basalt-hosted lithologies.

The WP samples did not perfectly cluster with any one biotope or lithology, nor did they cluster with all of the other shallow vent samples. This may reflect the complex lithology of the WP system as vent fluids pass through three distinct lithologic sequences: the mafic-derived Catalina Schist basement rock, the organic-rich Monterey Formation, and also Quaternary sediments (Woodring et al., 1946; Bebout and Barton, 1989; Grove et al., 2008). We hypothesize that this hybrid lithologic nature of WP generates a geochemical environment that selects for a diverse assemblage of microorganisms with similarities to a broad range of globally distributed hydrothermal communities, although this requires more detailed investigation. One limitation of our comparative analyses is a lack of replication in the pyrosequencing datasets we analyzed, including our own, many of which had only a single sample replicate from a given vent location or even vent field. These studies and our own have likely undersampled the variation in microbial diversity in these locations, as was suggested by our finding of a difference in the most abundant SOxB lineage in our two libraries. The lack of sample replicates meant that we could not perform direct site-to-site comparisons, so instead we compared across pooled categories of samples from multiple datasets. The reduced statistical power may have also reduced our ability to discern differences between and among hydrothermal systems (e.g., shallow vs. deep vents where we expected to observe greater differences due to the differences in temperature, pressure, light and geochemistry). Some recent studies have utilized a greater sampling effort to examine variability in deep-sea vent communities and found both intra- and inter-field variability in communities potentially driven by large scale differences in geological and geochemical processes, while in other comparisons, communities were indistinguishable (Flores et al., 2011, 2012). Ongoing work in the WP vent system will address this issue of biogeographic variation (Roussos et al., unpublished).

Overall, our results confirm the importance of both oxidative and reductive sulfur cyclers in the WP shallow water hydrothermal vent mats and demonstrate that biological sulfate reduction contributes sulfide to the WP system in addition to the abundant geologically derived sulfide. The high level of taxonomic diversity of both gamma- and epsilonproteobacterial SOxB lineages in the WP mats was somewhat surprising, as these lineages should all be competing for similar resources in these habitats, suggesting a degree of niche differentiation that is not fully understood at present. This study complements a recent report that used FISH and stable isotope probing to show that multiple groups of Gammaproteobacteria and a single coherent group of Deltaproteobacteria in the WP mat could be distinguished ecophysiologically based on their utilization of labeled acetate, ammonia and sulfate (Dawson et al., 2016). More broadly, our findings suggest that there is an underlying bacterial community structure that is shared among hydrothermal vent communities, although it also highlights that no two vent communities are identical, suggesting that variation in factors such as vent depth, biotope, lithology as well as perhaps other untested ecological or geological parameters combine to determine microbial habitation.

## Author Contributions

Conception or design of the work: PM, RH, VO, and JD. Data acquisition, analysis and interpretation: PM, NM, RH, and JD. Drafting the article: PM, and JD. Critical revision of the article: PM, NM, RH, VO, and JD. All authors have read and approved this submission.

## Conflict of Interest Statement

The authors declare that the research was conducted in the absence of any commercial or financial relationships that could be construed as a potential conflict of interest.

## Footnotes

1 http://nucleobytes.com/index.php/4peaks
