# Supplementary material for: Characterization of Chemosynthetic Microbial Mats Associated with Intertidal Hydrothermal Sulfur Vents in White Point, San Pedro, CA, USA
Source: Front Microbiol. 2016 Jul 27;7:1163. doi: 10.3389/fmicb.2016.01163 (PMC4961709; doi:10.3389/fmicb.2016.01163)
Supplement: Supplementary file 1 [file Image_1.PDF]

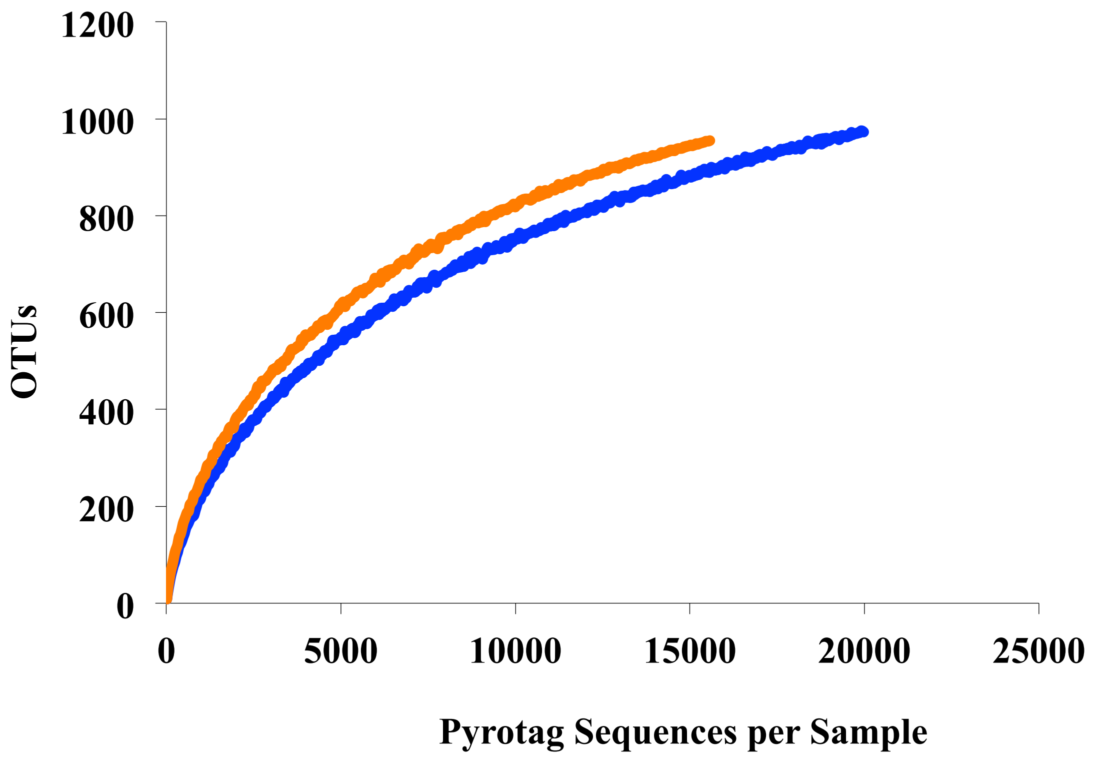

SUPPL. FIGURE 1. Rarefaction curves of WP microbial mat community pyrosequence datasets. OTUs calculated at 1% dissimilarity level. Blue = WP-1; orange = WP-2.

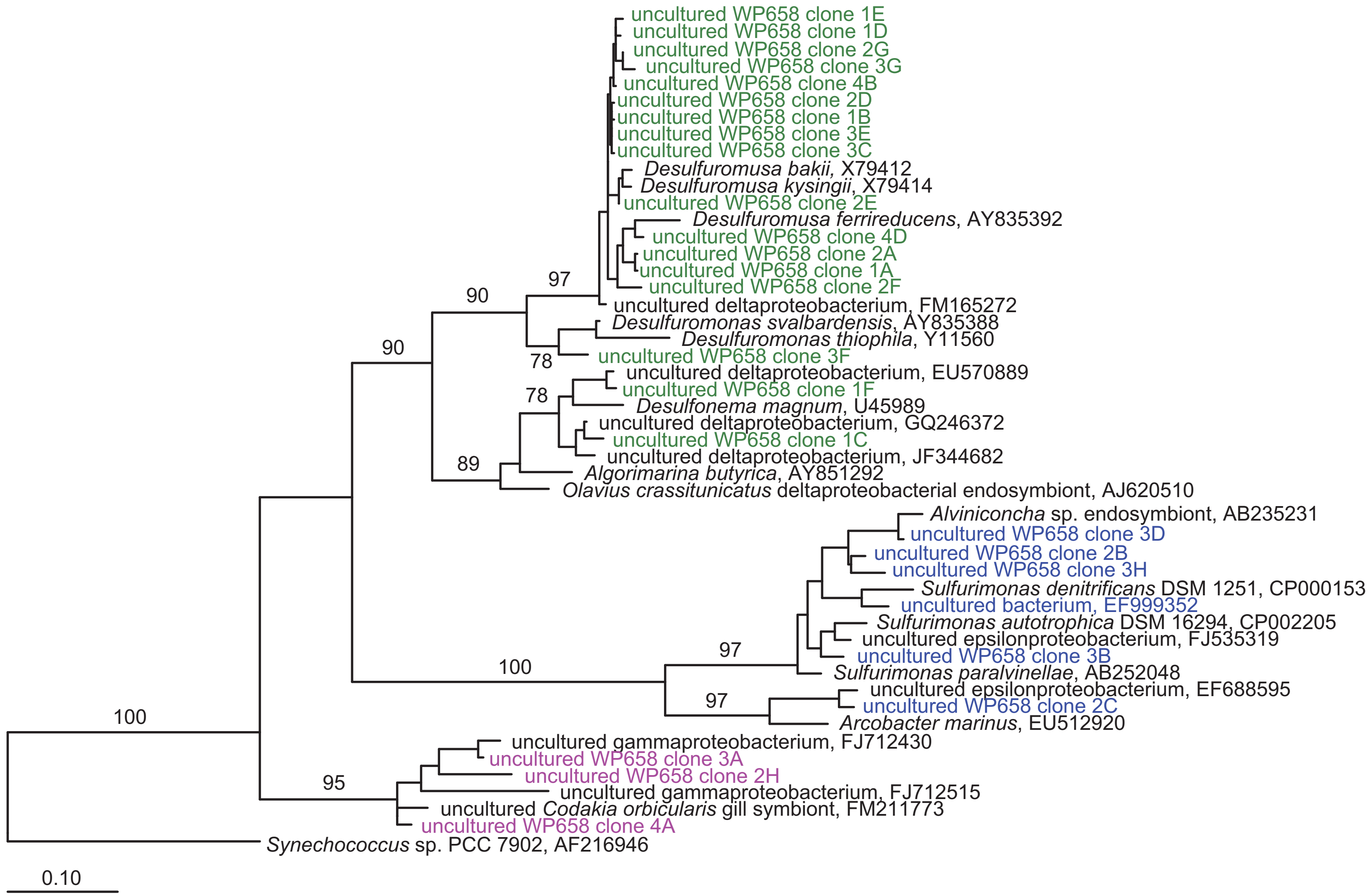

SUPPL. FIGURE 2. Unrooted maximum-likelihood dendrogram of 16S rRNA gene sequences amplified with DSS-658 reverse primer from WP microbial mat community, aligned with closest relatives (black) using ARB software. Colors indicate taxonomic groupings. Green = Deltaproteobacteria, blue = Epsilonproteobacteria and purple = Gammaproteobacteria. Values at nodes indicate >75% bootstrap support. Scale bar shows a 10% estimated difference in nucleotide sequence positions.
